# Tumor Models and Drug Targeting In Vitro—Where Are We Today? Where Do We Go from Here?

**DOI:** 10.3390/cancers15061768

**Published:** 2023-03-15

**Authors:** Marcus Krüger, Sascha Kopp

**Affiliations:** 1 Environmental Cell Biology Group, Department of Microgravity and Translational Regenerative Medicine, Otto von Guericke University, 39106 Magdeburg, Germany; 2 Core Facility Tissue Engineering, Otto von Guericke University, 39106 Magdeburg, Germany; sascha.kopp@ovgu.de

Cancer is one of the leading causes of death worldwide. It is therefore not surprising that numerous efforts are being made to combat cancer. Tumor models, used ex vivo to predict novel cancer markers, targeted therapies, or for drug development, are constantly evolving. Selecting the most appropriate model that best reflects the tumor system of interest is one of the major difficulties in cancer research.

Cells in the human body usually grow firmly embedded inside a structure-determining extracellular matrix and are surrounded by a biochemical microenvironment of extensive regulatory potential. This interaction not only determines the three-dimensional (3D) “natural” shape of organs and tumors but can also influence their functionality. Under standard cell culture conditions, adherent growing tumor cells form two-dimensional (2D) monolayers, a configuration that does not correspond to their actual situation in vivo. In solid tumors the concentrations of nutrients, oxygen, or metabolites decrease with increasing distances from blood vessels, affecting cell growth and metabolism. With the help of today’s technologies (3D cell cultures, bioprinting, vascular engineering, advanced bioreactors, smart biomaterials, stem cell differentiation, and microfluidics-based physiological platforms), it is now possible to control cells and their microenvironments more precisely, which in turn opens the door for the engineering of novel in vitro tumor models (Figure 1).

## 1. From 2D to 3D

More than 80% of ex vivo pilot studies for the development of new anticancer drugs are still conducted with 2D cell cultures [1]; however, these systems cannot adequately reflect the cellular responses in their microenvironments in vivo (such as tissues or tumors) and are of only limited prognostic value for the clinical efficacy of a novel drug. In fact, the absence of convincing preclinical models is one of the major reasons for the generally low success rate of drug development [2,3]. Far too often, promising drugs fail in the later stages of clinical development; therefore, it is advantageous to use the potential of more complex in vitro models to better mimic actual in vivo conditions [4]. Three-dimensional cell culture models represent a possible solution [5]. They can be classified into three categories: spheroid models, hydrogel-embedded cell cultures, and organ-on-a-chip systems.

## 2. From Spheroids to Organoids

Nowadays, tumor spheroids are used to study important processes, such as signal transduction, the differential gene expression of central and surface cells, metabolism, and differentiation in order to advance the knowledge on tumor growth [6]. In addition, they are used to investigate therapeutic issues that concern metabolic and proliferative gradients, such as the effects in chronically hypoxic tumor cells but also the role of cell–cell and cell–matrix interactions in radio- and chemotherapy [7,8]. Since tumor spheroids, such as tumors in vivo, exhibit permeability differences, active ingredients can be better investigated and possibly also more quickly excluded as therapeutic agents if the molecules do not reach all cells [9,10].

Heterocellular tumor spheroids (tumor organoids), generated by the co-cultivation of tumor cells with stromal cells, such as fibroblasts, or endothelial cells are an advanced and very complex 3D cell model [11]. Thus, an attempt is made to create a tumor-specific microenvironment in order to elucidate its involvement and key mechanisms in tumor development or tumor progression [12,13]. Tumor organoids show a more accurate replication of the tumor architecture and are ideally suited for drug development [14]. For this Special Issue, Rathje et al. [15] summarized the current knowledge on tumor organoids a with focus on colorectal cancer.

## 3. From Cell Lines to Patient-Derived Cells

Patient-derived tumor organoids (“microtumors”), grown from tumor tissue biopsies, stem cells, or organ-specific progenitor cells, enable personalized cancer therapy as they reflect the molecular and phenotypic properties of the underlying tumor tissue in a realistic manner [16]. Tumor organoids have already contributed to significant progress in personalized medicine. To illustrate this, Bae et al. [17] provided an update on the “promises and challenges” of patient-derived cancer organoids.

The biological heterogeneity observed in recurrent ovarian cancer might explain the strong differences in the clinical drug responses of these patients. Hoffmann et al. [18] modeled the interpatient tumor heterogeneity in druggable target expression and drug response on a patient-derived ovarian cancer spheroid model. The comparative testing of a variety of treatment options in the spheroid model resulted in more effective treatment than guideline-recommended therapies in 30% of patients.

Malignant mesothelioma is a rare malignancy that is also lacking in terms of an adequate number of in vitro models. Song et al. [19] have successfully established a syngeneic orthotopic model using malignant mesothelioma cells derived from an asbestos-induced *Cdkn2a*^+/−^; *Nf2*^+/−^ mouse. The model described represents genomic instability, and specific molecular targets for therapeutic or preventive intervention provide preclinical proof of concept. It is now straightforward to create a study cohort without the need to inject adenoviruses or asbestos.

## 4. From In Vitro to Ex Vivo

Ex vivo models are based on tissue extracted from organisms and cultured in a controlled external environment that resembles natural conditions. They are considered a compromise between in vitro and in vivo models and are more similar to human conditions, but also more complex.

Koch et al. [20] used a human ex vivo peritoneum model to mimic peritoneal carcinomatosis from colorectal cancer. In particular, this model allowed for the study of interactions between cancer cells and the tumor microenvironment. The results showed that matrix metalloproteinases (MMPs) were overexpressed during peritoneal colonization by colorectal cancer cells and that this overexpression could be prevented through the pharmacological inhibition of MMP-2 and MMP-9. MMP inhibition also significantly reduced peritoneal seeding in the functional primary culture model.

## 5. Updates on Models Mimicking Physiological Processes

It has been discussed that the cell biological program of epithelial to mesenchymal transition (EMT) is involved in both the development and progression of cancer [21]; however, experimental models for assessing this process in terms of its biological complexity remain limited. Peindl et al. [22] used a novel lung tumor tissue model with a preserved basement membrane to investigate EMT functions with respect to invasion across this membrane and drug resistance. They found evidence of an association between EMT and drug resistance in primary and secondary resistant cells carrying *KRAS^G12C^* or *EGFR* mutations. From their results, the authors conclude that EMT is a marker of drug resistance, rather than a trigger. Invasion may be favored by EMT but is more likely to depend on intrinsic factors. In addition, EMT was not detected in the center of invasive tumor nodules.

Melnik et al. [23] used a random-positioning-based metastasis model [24] to study the inhibitory effects of dexamethasone in more detail. The team of authors demonstrated that mechanical stress plays an important role in this in vitro model with follicular thyroid carcinoma (FTC) cells and that tumor cells not derived from metastases respond differently to this stress when compared to healthy or recurrent cells. Dexamethasone primarily restored a normal number of tight junctions in the FTC cells isolated from the metastasis, resulting in reduced detachment ability and thus the inhibition of “in vitro metastasis”.

## 6. Updates on Drug Delivery Techniques

Drug-loaded superparamagnetic iron oxide nanoparticles (SPIONs) appeared about 50 years ago [25]. Their inner iron core is magnetic, and the surrounding shell can be modified and adapted to obtain a nontoxic, biocompatible nanoparticle that can be loaded with chemotherapeutic agents. After intra-arterial application, SPIONs can be navigated to the tumor region by using an external magnet (magnetic drug targeting). Behr et al. [26] have now established an in vitro system with which to analyze the magnetic accumulation of drug-loaded SPIONs or SPION-loaded cells and their effects on tumor spheroids. For this purpose, spheroids were placed in a chamber system under the influence of a magnetic field and connected to a peristaltic pump to simulate blood flow. This allowed for the analysis of the magnetic accumulation and antitumor effects of magnetically targeted mitoxantrone as well as immune cells under dynamic conditions. The authors demonstrated that the accumulation mediated by the magnetic nanoparticles increased the antitumor effects and decreased the unspecific distribution of mitoxantrone as well as immune cells. According to the authors, it is the first non-handcrafted system that combines spheroids under dynamic flow with magnetic particles/cells and magnetic forces. Especially for nanomedical studies on magnetic transporters or cells, this application can bridge the gap between static experimental setups and in vivo experiments.

## 7. From Tumor-on-a-Chip to Human-on-a-Chip

The organ-on-a-chip (tissue chip) is a technological development that couples biology with microtechnology to mimic key aspects of human physiology, focusing on a tissue or organ of interest [27,28]. Human-on-a-chip approaches are more recent multiorgan systems that can be used to recapitulate the interactions between different tissues. Applied to cancer research, it may be possible to observe and understand the mechanisms of metastasis. In addition, these chips can be used to study the effects of cancer therapies directly on cancer and surrounding organs, offering new hope in personalized medicine [29].

In “From 2D Cultures to Organ-on-a-Chip Technology”, Foglizzo et al. [30] provided an up-to-date and comparative overview of existing advanced cellular models for preclinical drug testing up to the chip technology. The authors believe that animal testing on genetically modified mice and patient-derived xenografts will be partially replaced by in vitro studies in the future. 

With this Special Issue, we would like to provide an overview of the current developments in the field of in vitro tumor models. As Bae et al. [17] correctly noted in their conclusion, all cancer models have “pros and cons” when used as platforms for studying cancer biology; however, as such models are constantly revised and supplemented on the basis of underlying medical research, they also tend to reorganize knowledge and make it possible to identify previously unknown relationships. We thank all of the authors who contributed to this Special Issue. Ongoing research offers a brighter future for cancer treatment.

## Figures and Tables

**Figure 1:**
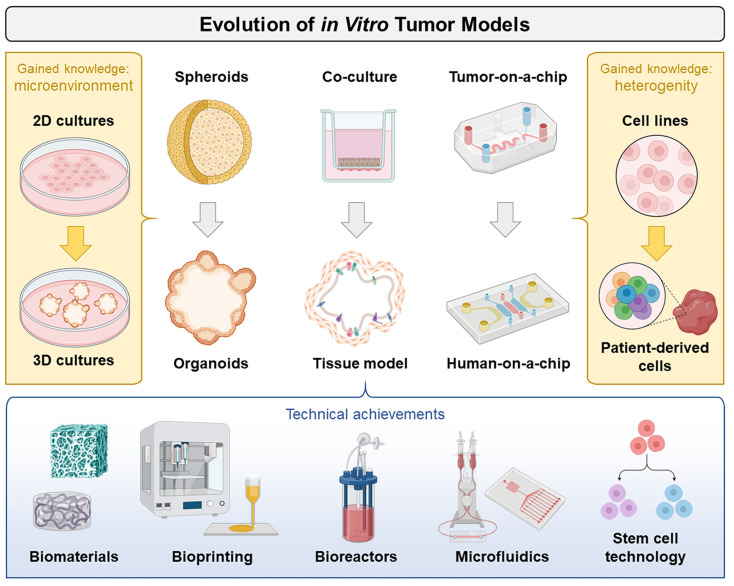
Illustrated overview of the evolution of in vitro tumor models. Increasing knowledge about the cell biology of tumors (yellow boxes) as well as technical progress with new methodological possibilities (blue box) are the driving forces for the further development of tumor models. The figure was created using elements from BioRender.com (accessed on 9 March 2023).
